# Health Workers’ Perspectives on the Outcomes, Enablers, and Barriers to the Implementation of HIV “Test and Treat” Guidelines in Abuja, Nigeria

**Published:** 2019

**Authors:** Solomon Odafe, Kristen A. Stafford, Aliyu Gambo, Dennis Onotu, Mahesh Swaminathan, Ibrahim Dalhatu, Uzoma Ene, Oladipo Ademola, Ahmed Mukhtar, Ibrahim Ramat, Ehoche Akipu, Henry Debem, Andrew T. Boyd, Aboje Sunday, Bola Gobir, Man E. Charurat

**Affiliations:** 1 Division of Global HIV/AIDS, Centre for Global Health, U.S. Centers for Disease Control & Prevention, Abuja, Nigeria; 2 Center for International Health, Education, and Biosecurity, University of Maryland School of Medicine, USA; 3 Division of Global HIV/AIDS, Centre for Global Health, U.S. Centers for Disease Control & Prevention, Atlanta, USA; 4 National AIDS & STIs Control Program, Federal Ministry of Health, Abuja, Nigeria

**Keywords:** HIV/AIDS, Treat All, Test and Treat, Guidelines, Health workers’ perceptions, Barriers, enablers

## Abstract

We evaluated health workers’ perspectives on the implementation of the 2016 HIV “Test and Treat” guidelines in Nigeria. Using semi-structured interviews, qualitative data was collected from twenty health workers meeting inclusion criteria in six study sites. Data exploration was conducted using thematic content analysis. Participants perceived that the “Test and Treat” guidelines improved care for PLHIV, though they also perceived possible congested clinics. Perceived key factors enabling guidelines use were perceived patient benefits, availability of policy document and trainings. Perceived key barriers to guidelines use were poverty among patients, inadequate human resources and stock-outs of HIV testing kits. Further improvements in uptake of guidelines could be achieved by effecting an efficient supply chain system for HIV testing kits, and improved guidelines distribution and capacity building prior to implementation. Additionally, implementing differentiated approaches that decongest clinics, and programs that economically empower patients, could improve guidelines use, as Nigeria scales “Test and Treat” nationwide.

## Introduction

HIV/AIDS continues to be a major public health disease accounting for 35 million deaths across the world. In 2016 alone, there were 1.8 million new HIV infections and 1 million deaths worldwide. At the end of 2016, there were approximately 36.7 million people living with HIV (PLHIV) including 2.1 million children worldwide. Africa remains the worst hit continent by HIV/AIDS, accounting for 25.6 million (70%) of PLHIV globally [1]. Since the introduction of Antiretroviral Therapy (ART), there has been a 43% reduction in annual deaths due to HIV/AIDS [2]. By mid-2017, though there were approximately 20.9 million PLHIV including 120,000 children receiving lifesaving ART, this number represents only 57% of the total PLHIV globally [3]. The United Nation General Assembly (UNGASS) has committed to ending the AIDS epidemic by 2030. In June 2016, UNGASS signed on to a political declaration of the 90–90-90 strategy, with the objectives to identify 90% of individuals who are HIV positive, treat 90% of all identified individuals who are HIV positive and achieve viral suppression in 90% of those on treatment by 2020 [4]. A key strategy for achieving epidemic control is increasing access to lifelong ART [2]. In order to improve access to ART, in June 2016, the World Health Organization launched the implementation of the “Test and Treat” guidelines, which recommend the immediate treatment of all individuals tested HIV positive [1].

The Federal Republic of Nigeria is in West Africa and has a population of 190 million people [5]. The country has one of the largest HIV burden in the world with an estimated 1.9 million HIV infected persons [6]. In 2016, there were about 220,000 HIV new infections and about 160,000 new AIDS-associated deaths in Nigeria. Only about 30% of PLHIV are on antiretroviral medication [6]. Subsequent to achieve the UNAIDS strategic treatment target within the 90–90-90 strategy, the President’s Emergency Plan for AIDS Relief (PEPFAR) in Nigeria piloted the “Test and Treat” approach in clinical management of PLHIV in 32 local government areas (LGAs) in Nigeria between March 2016 and December 2016, including in the capital city, Abuja [7]. In December 2016, the government of Nigeria through the Federal Ministry of Health (FMOH) published the guidelines to “Test and Treat” all individuals identified as HIV positive in the country to help improve access to lifesaving ART for all HIV infected persons, and for those newly diagnosed with HIV, to start ART within two weeks of diagnosis [8]. However, there are currently shortages of human resources across all cadres of health staff in Nigeria due to outmigration of health care workers, poorly motivated health workforce and mal distribution of health staff [9]. Consequently, there have been concerns that the new HIV treatment guidelines requiring that all HIV positive individuals be treated will increase the burden on the already overstretched health system and potentially compromise care for PLHIV. Additionally, as HIV treatment program managers scale up implementation of the “Test and Treat” approach nationally, exploring health workers’ perspectives on potential enablers and barriers to implementation is useful for devising effective strategies for nationwide scale-up of the approach. Thus, this study aimed to understand health workers’ perspectives on the implementation of the “Test and Treat” guidelines on the HIV treatment program in Nigeria, specifically their perceptions of the guidelines’ impact on patient care and outcomes for PLHIV and of potential enablers and barriers of effective guideline implementation, with an eye to use those lessons to inform national implementation of “Test and Treat”.

## Materials and Methods

### Study area and design

This was a qualitative study of health workers’ perceptions using an in-depth interview approach. By utilizing an in-depth interview approach, the researchers were able to explore participants’ understanding and perceptions on the implementation of “Test and Treat” guidelines at their various clinics [10].

### Epistemological approach

The epistemological approach for this study is entrenched in an interpretivist or constructionist paradigm [11]. The interpretivist paradigm believes that people construct their own understanding of reality from their diverse personal experiences [12]. In alignment with that ideology, during interviews participants were required to draw meaning with respect to how the introduction of the new “Test and Treat” guidelines changed their practice and impacted the lives of their patients. The theoretical basis for this study is that health workers’ perceptions are of critical importance in determining the extent and success of the implementation of the “Test and Treat” guidelines on patient care because they play the role of the care provider. Additionally, they are experts with capacity to judge improvement or lack of it in management of PLHIV [13].

### Study setting

The study was conducted in Abuja Municipal Area Council in the Federal Capital Territory (FCT), Abuja, Nigeria. Abuja is the administrative capital of Nigeria and has an estimated population of 3,841,079 [14]. The FCT Abuja has an HIV prevalence of 5.8%, with approximately 80,000 individuals living with HIV in the city [15]. As of June 2017, there were approximately 43,000 HIV-positive individuals receiving ART in FCT Abuja [16].

### Sampling approach

Health worker interviews for the study were conducted at six hospitals between February and March 2018 within LGAs in FCT Abuja where the “Test and Treat” guidelines had been piloted between March and December 2016 [7]. A purposeful sampling was used to select participants for the interviews within the study at each site [17]. In purposeful sampling technique, predefined criteria are used to determine selection of participants based on the requirements of study objectives [18]. Six sites were sampled as part of larger evaluation of Test and Treat, and then all health workers meeting inclusion criteria at each site were interviewed. Table 1 summarizes the predefined criteria, including inclusion and exclusion criteria, for participants in interviews within the study. Twenty health workers that met inclusion criteria at the selected sites were interviewed for the study.

### Data collection methods

The individual face-to-face in-depth interview methodology was best suited for the study because of its inherent ability to explore individuals’ perspectives and provide detailed information on behavior and related social context [10]. All interviews were conducted at the study site, in environments familiar to the participants. Health workers usually work in very busy clinics, and in order to guarantee privacy, the researchers used available empty office spaces. The researchers sought permission from each participant before digitally recording interview sessions. Additionally, a record of important statements made, or activities observed during the fieldwork was kept as notes from the sessions.

### Study instrument

The study used an interview guide specially developed for the study, guided by a previous study evaluating guidelines among health workers in Kenya [19]. All interviews were conducted in English. The interview guide covered knowledge about the health workers’ experience using the “Test and Treat” guidelines, the care and outcomes of patients since the introduction of the “Test and Treat” guidelines, and perceived enablers or barriers to using the guidelines in clinical care. The interview guide was piloted through three health workers in different departments at a comprehensive HIV treatment center in Abuja.

### Data analysis

The researcher utilized thematic content analysis to explore the data collected during the interviews [20]. After the interviews, participants’ responses were transcribed verbatim and the lines of text numbered [21]. All transcripts were anonymized to ensure confidentiality. The transcription process was followed by coding of the transcripts. Thereafter, the descriptive codes were identified, and analytic codes were created. Afterward, major themes were identified. A codebook containing both descriptive and analytic codes that related themes was used to guide the development of the study discussion and conclusion. To ensure trustworthiness, the researchers reviewed transcripts with respondents to ensure the content captured what participants intended and used appropriate quotes during reporting [22].

### Ethical consideration

The study protocol was reviewed and approved by National Health Research Ethics Committee of Nigeria. This activity was also reviewed in accordance with the Centers for Disease Control and Prevention (CDC) human research protection procedures and was determined to be research, but CDC investigators did not interact with human subjects or have access to identifiable data or specimens for research purposes. An informed consent was obtained from all participants interviewed. All participants were informed of their rights to voluntary participation [23]. Information provided during the interview did not include personally identifiable information and only aggregated data and unlinked quotes were used for the study reports. All participants’ names were replaced with numbers in the transcripts, during analysis and reporting [24].

## Results

A total of 20 health workers were interviewed in the study, comprising 8 medical doctors, 5 nurses or adherence counsellors, 4 laboratory scientists, 2 monitoring and evaluation (M&E) officers and one data analyst. From the qualitative analysis of their interview responses, we identified four major themes of importance describing (1) the perceived benefits of the guidelines change on clinical practice, (2) the perceived disadvantages of the guidelines change on clinical practice, (3) perceived enablers/ barriers to guidelines change and (4) recommendations for improvement in guidelines implementation. Table 2 summarizes the themes, and codes from the analysis of the transcripts from the interviews.

### Perceived benefits of the guidelines change on clinical practice

The health workers interviewed in the study generally believed that the “Test and Treat” approach has been of tremendous help in improving patient care. Perceived benefits experienced due to the introduction of the new national “Test and Treat” guidelines include early initiation on ART. Early treatment start means doctors no longer wait until patients’ health deteriorates before commencing treatment. In addition, health workers also perceived that the new approach may have helped in retaining more pre-ART patients in care:

#### Participant (P)16 (adherence counsellor):

“It is helping also to initiate treatment early and of course help to retain patients in care before starting treatment. In the past, we delayed patient treatment and they go away”

Additionally, starting treatment immediately after testing means the need for repetitive follow-up visits, delay in treatment start and risk of losing the patient between the time of test and time of treatment initiation are reduced.

#### P2 (Adherence counsellor):

“We take them round and round, they go and come before they receive their treatment but with the test and treat [“Test and Treat”] we are able to give them the treatment and they go and they are happy for it”

#### P1 (Doctor):

“The initial loss after diagnosis has greatly reduced. You know the way our society is structured, sometimes when they go, they never come back. They end up in the churches and some in other places.” Another important benefit of the “Test and Treat” approach was a perception of improvement in patients’ health outcomes. Health workers reported that since the start of the guidelines, there have been increased testimonies from patients that they are doing much better:

#### P1 (Doctor):

“The testimony we have is some of them returning to say (…) Doctor since we started this drug, I am getting much better”

#### P13 (Nurse):

“There has been great advantage, because it is a disease we cannot cure. But if you are starting treatment immediately it makes the patient live healthier and longer”

Health workers perceived that application of the previous HIV treatment guidelines was very stressful for the patients. It required multiple clinic visits, frequent blood draws for CD4 investigation, and long hours of repeated counselling sessions before treatment was initiated. The process required multiple interactions between patients and health workers, some of which could be perceived as unnecessary, time wasting and stressful. However, the implementation of the new guidelines has simplified the process and reduced the stress on the patient. Furthermore, health workers can obtain complete records from patients because all activities are concluded on the same day:

#### P7 (Nurse):

“Okay, the promptness of it. And then the reduction in the stress on the part of the patient. And then even on the side of the health workers, there are advantages because it helps us get all the recordings the same day.”

Participants perceived that the early initiation of treatment under the new guidelines is giving health workers hope for a better recovery for the patient:

#### P2 (Adherence counsellor):

“On our own side (health worker) it has increased our hope of recovering the patient.”

#### P11 (M&E officer):

“So, that fear [of dying] has been taken away. Immediately, I am tested, I am started on drugs.”

### Perceived disadvantages of the guidelines change on clinical practice

Respondents identified some drawbacks due to the implementation of the new “Test and Treat” guidelines. Health workers reported that a major success from the previous guidelines was a three weeks’ treatment preparation class in which patients received an intensive education and counselling session, which stood out from other pre-treatment requirements as being useful and well-liked. The treatment preparatory class not only educated the patients but also created a platform for interaction between patients and health workers and patients with other patients. However, with the implementation of the new guidelines the treatment preparatory class is no longer available:

#### P11 (M&E officer):

“Unlike the previous guidelines whereby you get to know your status and you are taken on a three weeks treatment preparatory class for you to get an understanding of all it takes to be positives and living with the virus. But now you get tested immediately and you are started on drugs (…) they might not really appreciate what it takes to be living with the virus.”

There are also concerns that the new guidelines have increased workload because under new guidelines standards, all PLHIV not currently offered treatment must be offered treatment, and all newly diagnosed PLHIV must start treatment immediately. Respondents explained that since the implementation of the “Test and Treat” guidelines, the number of patients eligible to start treatment has increased without a corresponding increase in the number of health workers providing health care. Consequently, they perceive that clinics have been crowded, increasing patient waiting time. Moreover, health workers perceived that patients are often wary of coming to clinics if they will spend a long time waiting.

#### P14 (Doctor):

“Because the test and treat [“Test and Treat” guidelines] have increased the workload. So making the clinic a busy one for the clinicians.”

#### P1 (Doctor):

“They are not waiting for you to waste their time anymore.”

All respondents agreed that they perceive a reduction in the initial patient loss between positive testing and treatment initiation. However, there was a division of opinion on the perceived rate of attrition due to lost to follow up after patients started ART. The findings appear to vary by treatment centre; some health workers interviewed perceived that there has been no change in lost to follow up:

#### P10 (doctor):

“I wouldn’t say that has changed because most of the causes of lost to follow up in our environment is financial problems basically. So, I think it’s about the same.”

Whereas, some health workers believed lost to follow up has increased since the implementation of “Test and Treat”:

#### P6 (doctor):

“But, talk about lost to follow up, I think we have more lost to follow-ups since we started test and treat [“Test and Treat” guidelines].”

Some other health workers perceived that there might actually be a reduction in lost to follow up with the commencement of “Test and Treat”:

#### P9 (doctor):

” There is no lost to follow up again because they already know they are on drugs (…) Lost to follow up is reduced to the barest minimum if there is any.”

### Perceived enablers affecting guidelines use

Health workers report that an important enabler in implementation of “Test and Treat” is the training on the use of the guidelines. Participants explained that training gave them the capacity to utilize the guidelines. Therefore, all health workers trained were willing to start using the guideline after receiving training:

#### P1 (Doctor):

“They educated every person that participated actively in this program. You understand? And once they understood, it was easy for them to follow up.”

Participants further explained that there has been a variety of factors enabling the use of the “Test and Treat” guidelines at the various hospitals. The factors enabling guidelines use include the passion and interest of the health worker in caring for patients:

#### P5 (Doctor):

“Okay, number one for me is the passion of the health workers.”

Another important enabler to the use of the guidelines was the availability of tools required to work, such as medications, test kits, job aids, and the written guidelines themselves in most clinics. Respondents further explained that health workers were likely to implement the “Test and Treat” guidelines in hospitals where the staff have sufficient supply of test kits and drugs. Furthermore, the availability of job aids and guidelines makes it easy for referencing and utilization of policy direction in the guidelines.

#### P3 (M&E Officer):

“Hmmm, (…) the availability of the tools (…) they also brought job aids, it has helped (…) at least to refresh on the knowledge from the training.”

#### P6 (Doctor):

“Because if we didn’t have drugs that will mean the clients will come and we will not be able to start their treatment.”

Respondents further explained that good management support and regular government supervisory visits are added motivation for implementing the guidelines.

#### P11 (M&E Officer):

“Since the supervisory visit is regular, they give you an update or give you a step-down and say this is how it should be or do it this way.”

### Perceived barriers affecting guidelines use:

Participants highlighted several factors that made it difficult to implement the “Test and Treat” guidelines. However, the most important perceived barrier that cut across nearly all study sites was patient poverty. The patient’s inability to pay for transportation to site or baseline investigations was a major barrier to the implementation of the “Test and Treat” guidelines.

#### P1 (Doctor):

“A patient comes in and obviously you just see anemia, why are you not eating what was recommended? They will say they don’t have money.”

#### P2 (Adherence counsellor):

“So out of about five of them I saw this morning only one assured me that he will come to the meeting the other ones said ‘Mummy no transport [money for transportation to the meeting].”

Another barrier identified was stock out of test kits, which makes it impossible to test new patients for HIV in few hospitals. Some health workers also cited a lack of sufficient copies of guidelines for staff review was also a barrier to effective implementation.

#### P4 (Nurse):

“Though sometimes we run short of test kits. It is important that everything we need to work is available. Sometimes there we are short of supplies.”

#### P13 (Doctor):

“Yes, another barrier is if you don’t have enough copies of the guideline for everybody (health workers) to have. In this facility doctors change frequently so the two or three copies they brought is not enough.”

Additionally, a lack of a dedicated space to provide treatment for tuberculosis, which is considered the most common opportunistic infection among PLHIV in Nigeria, and more generally a lack of integration of TB care with HIV care, may constitute a barrier to ensuring comprehensive care among PLHIV in some clinics.

#### P11 (M&E Officer):

“We have a challenge with the DOT [Directly Observed Therapy for TB treatment] clinic, our TB/HIV integration is not doing so well.

Furthermore, the level of training may be a barrier because younger doctors do not have enough information about the guidelines and may require more training to come up to speed about the change.

#### P6 (Doctor):

“The level of training may be a barrier, we have younger doctors, house officers, youth corpers, they are here for just one year.”

Finally, respondents explained that inadequate human resources, specifically, inadequate numbers of health staff, is a major barrier to implementation of the new guidelines. As the number of patients continue to increase, respondents felt that there needs to be a corresponding increase in number of health workers to ensure quality of care is not compromised.

#### P14 (Doctor):

“Then another barrier is manpower. Because the test and treat [“Test and Treat”guidelines] has increased the workload. So, making the clinic a busy one for the clinicians.”

### Recommendations for improvement in guideline implementation

Interviewed participants provided some very important ideas for improving on some of the barriers identified during the study, in order to improve and scale up implementation of the “Test and Treat” guidelines nationwide. A major recommendation was for the government to introduce some form of financial assistance scheme to support patients who could not afford to transport themselves to clinics or pay for required laboratory investigations.

#### P12:

“Some of them have problem of coming here with their TP [Transportation] … for some of them feeding problems and all that. (…) At each of their visits if we can give a little thing, a little token to help them so they can come back next time for their visit.”

The “Test and Treat” guidelines pilot implementation team could help improve and scale up implementation through advocacy to the government on the need to provide additional clinical staff to support the growing number of patients at the clinics. The implementation team can also advocate for laboratory staff at the laboratory to reduce processing time for the additional viral load tests.

#### P14 (Doctor)

“I think there is still need for more advocacy for some clinicians.”

#### P15 (Data Analyst):

“It is to involve the lab. We need to get them together and tell them the implications of not starting the patients on treatment.”

Another area that a government intervention will help with is in improved support to assist TB/HIV integration to ensure comprehensive care for PLHIV.

#### P11 (M&E Officer):

“So, I want to make a suggestion, if we can find a way to help to revitalize the DOT section of the hospital.”

Another area is ensuring more government support to sites through supervision to the hospitals and provision of sufficient copies of the guidelines to all HIV clinics including more concise pocket versions of the guidelines.

#### P13 (Doctor):

“Yes, another barrier is if you don’t have enough copies of the guideline for everybody. There could be pocket versions of the guidelines that will make it easy to carry about.”

Lastly, hospitals could implement a more differentiated service delivery approach that takes services into the communities to decongest the clinics.

#### P14 (Doctor):

“It has helped to decongest the clinic. For example, some of the patients don’t need to come to the clinic again they just go to the community pharmacy to pick up their drugs.”

## Discussion

AAmong health workers, the implementation of the “Test and Treat” guidelines in Nigeria was perceived to have significantly changed clinical practice for care of PLHIV, bringing about reduction in delays before treatment start, reduction in stress and increase in hopes among health workers for a better outcome. Additionally, because all events leading to treatment initiation are completed on the same day, there are more complete records for patient follow-up. Among health worker respondents, the perception was that health workers were willing to implement the new guidelines because of perceived health benefits of earlier ART initiation in their patients. The study finding was consistent with reports by Nzinga et al and Odhiambo et al. Both studies observed that knowledge of the benefits from implementing guidelines was a major motivational factor to utilizing guidelines [19,25]. The study results suggest that new public health initiatives such as guidelines or policy changes require significant health worker education to increase the chances of successful implementation. Specifically, in the Nigerian context, the results suggest that expansion of “Test and Treat” nationally should be coupled with health worker education about the health benefits of starting treatment early.

Furthermore, health workers perceived that the introduction of the “Test and Treat” guidelines led to improvements in treatment outcomes. This perception was consistent with findings previously reported in the literature [26–28]. However, there was a perception that the “Test and Treat” guidelines alone cannot resolve lost to follow up in patients on ART. Health workers perceived that there were multiple factors responsible for lost to follow up, topmost among them was financial difficulties, making it challenging to afford transport to clinic and payment for investigations. This finding was consistent with reasons for attrition identified by patients in previous reports [29–31]. Maskew et al., in their study in South Africa, conducted among 182 HIV positive patients who missed follow up appointments, and contacted through telephone calls, reported that a majority of their patients cited financial difficulties and inability to pay for transport cost as major reasons for missed appointments [29]. Consequently, there are no guarantees that starting treatment early may lead to a reduction in lost to follow up. The implication of this finding is that program managers must adopt strategies that empower the clients to meet up with clinic appointments and treatment approaches that take services closer to communities to reduce lost to follow up [32–34].

Factors previously identified as enablers of guidelines use included collaboration between facilities, peer support, and provider characteristics such as higher level of education, good commitment, and knowledge of guidelines [35]. The major factors identified in the literature that serve as barriers to treatment guidelines implementation included human resource gaps, lengthy guidelines, and inadequate supplies of medicines and commodities [35]. This study found that training, availability of tools, government supervisory visits, hospital management support and health workers passion and interest enabled guideline use. Identified barriers in this study included patient poverty, inadequate human resources, and stock-outs of tools, guidelines, and test kits. Thus, the identified enablers and barriers to guidelines use were comparable to those previously reported [35]. The implication of these findings is that program managers must ensure that clinics have staff strength adequate for patient load, ensure adequate supervisory visits, provide adequate supplies of test kits and guidelines and implement innovative approaches that decongest clinics in order to improve the impact of the implementation of the “Test and Treat” guidelines. Each of these will need to be addressed as Nigeria scales up “Test and Treat” nationally.

Focusing specifically on patient poverty as a main barrier to “Test and Treat” implementation, the study revealed that patients’ lack of capacity to pay for baseline investigation or transport services to clinics limited their ability to access treatment services. The results re-affirm the concept of a wider set of determinants for health and the important effect of socioeconomic factors in determining the health of individuals [36–38]. The study demonstrates that the presence of social support systems can influence an individual’s health-seeking behaviors [39]. The study results suggest that to improve retention among the higher number of PLHIV on ART inherent in the “Test and Treat” approach, HIV programs in Nigeria may need to incorporate social support safety nets to help indigent patients.

Health worker respondents perceived that the challenges of patient access could be mitigated by implementation of patient empowerment schemes that increase patients’ skills and empower them economically to provide for themselves and their households. An economically empowering scheme implemented in Kenya enabled HIV-positive persons through capacity building and provision of soft loans to become financially sufficient [40]. A similar approach in Nigeria may contribute to improved attendance at clinics as “Test and Treat” expands nationally. The study also revealed that many patients still come from distant communities to access HIV care and treatment in big cities. This finding suggests that implementing community-based HIV treatment models that take services into communities using various approaches such as mobile clinics, community adherence clubs and community drug dispensing outlets may reduce the need for patients to travel far and further improve adherence to treatment [32,34,41].

Focusing specifically on inadequate health staff as a main barrier to “Test and Treat” implementation, the government could resolve this challenge by scaling up a variety of approaches including the use of six-month prescriptions that give stable patients up to six months’ drug refills at a time, drastically reducing the frequency for clinic visits [42]. Moreover, the current treatment guidelines in Nigeria supports the use of multi-month scripting [8]. Additionally, Nigeria already has a policy on task shifting in HIV care and treatment, and reports suggests that these approaches are currently being implemented in Nigeria and are yielding good results in maintaining PLHIV on treatment [43]. Therefore, scaling up approaches such as task shifting and task sharing that build the capacity of nurses to provide clinical care and allow nurse prescription of ART could further expand the existing human resource capacity for HIV services in Nigeria [44,45].

The strengths of the study included the rigorous process to ensure conformity with established qualitative research and ethical standards. The use of an interpretivist epistemological approach, reinforced by a social constructivist standpoint, allowed the authors to obtain the perspective of health workers on “Test and Treat” [10]. Additionally, the use of the interview guide and appropriate probes ensured that all participant were measured using similar standards [46], thus reducing measurement and social desirability biases. To mitigate the role of positionality, the researchers ensured that their views were bracketed by identifying their own preconceived assumptions and setting them aside during the interview process [47,48]. Lastly, whereas previous evaluation focus on perspective of patients [29–31], this study obtained the perspective of health workers on the implementation of a new health policy.

A major limitation of the study was the authors’ inability to triangulate health workers’ perceptions of patient reactions to the guideline changes with actual patient perceptions because patients’ perspectives were not collected. A qualitative design that obtains the perspectives of the patients themselves is recommended as a potential area for future studies [49,50]. Another limitation of the study was the generalizability and transferability of its findings, because the study was conducted in only one state in the country and in selected sites [51]. Notwithstanding the limitations, our study offers important information that can guide larger public health evaluations and provide insights on how to maximize acceptability and implementation of the “Test and Treat” guidelines nationally.

## Conclusions

According to participating health workers, the introduction of the “Test and Treat” guidelines in Abuja, Nigeria improved access to skilled HIV care and treatment services for PLHIV and were perceived to improve patients’ overall health. Factors enabling the implementation of the “Test and Treat” guidelines were training of health workers on guideline use, the passion of health workers to see patients get better, and the availability of tools, government supervisory visits and good hospital management support. The identified barriers to successful guidelines implementation were poverty, inadequate human resource at clinics, and stock-outs of test kits. Study findings suggest that the effective implementation and scale up of the “Test and Treat” guidelines, with the intention of improving PLHIV clinical outcomes, could be further enhanced if the government provided support for indigent patients, more health worker staff, adequate supervisory visits, adequate supplies of test kits, and copies of guidelines. In addition, implementing innovative approaches that decongest clinics by providing more services in communities would enhance effective implementation of the guidelines. In these ways, the “Test and Treat” approach can be effectively expanded nationally, thus giving PLHIV in Nigeria timely access to comprehensive HIV care and treatment.

## Figures and Tables

**Table 1:** Study inclusion and exclusion criteria for participants in interviews.

Criteria	Category
Inclusion criteria	Health worker, who may be a doctor, pharmacist, nurse, laboratory scientist, record office clerk, or data analyst or program manager; who is working at the HIV treatment program and has served three or more years in the HIV program.
Exclusion criteria	Nonconsenting health workers

**Table 2:** Coding scheme from thematic content analysis.

Main themes	Codes
Perceived benefits of the guidelines change on clinical practice	1.1 No delays before treatment start
	1.1.1 Reduced pre-treatment losses
	1.2 Improved perceived patient outcomes
	1.2.1 Reduced pre-treatment losses
	1.3 Reduced stress on the patient
	1.4 Complete patient records
	1.5 Removes patients fears and gives health workers hope
	1.6 No more repetitive visits prior to starting treatment
Perceived disadvantages of the guidelines change on clinical practice	2.1 Reduced time for patient education prior to starting treatment
	2.2 Increased workload for health workers
	2.3 Overcrowded clinic & increased waiting
	2.4 Increased on-treatment lost to follow up
	2.4.1 Factors responsible for attrition
Perceived enablers/barriers affecting guidelines use	3.1 Enablers
	3.1.1 Training on guideline use
	3.1.2 Health worker passion and interest
	3.1.3 Availability of tools: Job aids, guidelines
	3.1.4 Government supervisory visits
	3.1.5 Management support
	3.2 Barriers
	3.2.1 Poverty of patients
	3.2.2 Stock-outs of test kits
	3.2.3 Insufficient guidelines copies for health workers
	3.2.4 Challenges in TB/HIV integration
	3.2.5 Inadequate training on new guidelines for junior doctors
	3.2.6 Inadequate human resources for implementation
Recommendations for improvement in guideline implementation	4.3 How to improve perceived barriers
	4.3.1 Financial Assistance
	4.3.2 Improved patient education
	4.3.3 Advocacy for more clinicians and laboratory staff
	4.3.4 Improved support for TB/HIV services
	4.3.5 Provision of sufficient guidelines copies
	4.3.6 Implement differentiated service delivery approach to treatment
